# Acute Radial Artery Thromboembolic Occlusion During Transcatheter Aortic Valve Replacement: A Case Report and Review of Management Strategies

**DOI:** 10.7759/cureus.64307

**Published:** 2024-07-11

**Authors:** Shubam Trehan, Gaurav Bector, Gurjot Singh, Aayush Jain, Nadish Garg

**Affiliations:** 1 Division of Cardiology, Memorial Hermann Southeast Hospital, Houston, USA; 2 Division of Internal Medicine, Memorial Hermann Southeast Hospital, Houston, USA; 3 Division of Cardiology, Memorial Hermann Pearland Hospital, Pearland, USA

**Keywords:** thromboembolic occlusion, transcatheter aortic valve replacement, post-tavr complications, severe chest pain, radial artery occlusion, tavr, severe aortic stenosis

## Abstract

Acute limb ischemia requires prompt diagnosis and treatment. Thromboembolic events are common, especially in patients with multiple risk factors. This case report describes a rare complication of transcatheter aortic valve replacement (TAVR) involving thromboembolic occlusion of the radial artery and highlights the risk of embolic complications during TAVR. While TAVR is minimally invasive and preferred for high-risk patients, it carries the risk of complications such as paravalvular leakage and cerebrovascular events. Prompt recognition and management are crucial. Various mechanisms, including catheter manipulation and altered hemodynamics, contribute to embolic risks during TAVR. Awareness and management of rare embolic complications during TAVR are essential. Further research is needed to prevent these complications and improve patient outcomes.

## Introduction

Acute limb ischemia is a medical emergency that requires prompt diagnosis and treatment to prevent permanent tissue damage and preserve limb function. Thromboembolic events are frequently the cause, especially in individuals with multiple risk factors such as untreated hypertension, smoking, and advanced age [1]. Historically, surgical thrombectomy has been the primary treatment for acute thromboembolic upper limb ischemia. However, newer treatment modalities have emerged, enhancing the management of this condition. Endovascular techniques, such as catheter-directed thrombolysis and percutaneous mechanical thrombectomy, offer less invasive alternatives with shorter recovery times [2].

Aortic stenosis (AS) is the most common valvular heart disease in developed nations, affecting around nine million people worldwide [3]. Its prevalence increases with age, affecting up to 10% of those over 80 years old [4]. Severe AS, defined by an aortic valve area of less than 1.0 cm², occurs in 2-4% of individuals aged 75 years and older [5]. Due to its minimally invasive approach and reduced recovery times, transcatheter aortic valve replacement (TAVR) has become the preferred treatment for severe AS, especially in patients who are high-risk candidates for surgical aortic valve replacement (SAVR) [6].

Despite its advantages, TAVR is not without complications. Paravalvular leakage (PVL), conduction disorders, and cerebrovascular events are among the known risks associated with the procedure, occurring in approximately 1% to 5% of cases [7]. Cerebrovascular complications, such as stroke and transient ischemic attacks (TIAs), are more commonly discussed in the literature due to the high morbidity associated with these events [8]. However, acute thromboembolic occlusion of the radial artery during TAVR is a rare and underreported complication [9].

Several case reports and studies have highlighted the occurrence of thromboembolic events following TAVR. For instance, a case report described acute limb ischemia following the administration of protamine sulfate in a post-TAVR patient, underscoring the thromboembolic risks associated with the procedure [6]. Additionally, other reports have documented complications such as acute limb ischemia resulting from TAVR valve migration and endovascular retrieval of embolized devices causing acute limb ischemia [10].

Peripheral artery disease (PAD) is another factor that has been associated with worse outcomes after TAVR, including increased limb ischemic events [10]. PAD complicates the procedure by posing challenges in vascular access, as patients often have calcified, narrowed, or tortuous arteries. This makes catheter and device navigation more difficult, increasing the risk of vascular complications such as dissection, perforation, and embolization. To mitigate these risks, meticulous intraoperative monitoring and management are essential. Preoperative assessment should include detailed imaging studies like computed tomography angiography (CTA) to map out vascular anatomy and identify potential challenges. During the procedure, advanced imaging techniques such as intravascular ultrasound (IVUS) and fluoroscopy can guide catheters and ensure precise valve deployment. Postoperatively, patients with PAD require close monitoring for signs of limb ischemia and other vascular complications, with early intervention being crucial for optimal outcomes. A multidisciplinary approach involving cardiologists, vascular surgeons, and interventional radiologists can enhance management, combining expertise and advanced techniques to improve the safety and efficacy of TAVR in patients with PAD, ultimately leading to better long-term outcomes.

## Case presentation

A 79-year-old African-American female with a history of severe aortic stenosis (AS), lung cancer, and infrarenal abdominal aortic aneurysm (AAA) presented for TAVR. The patient presented with symptoms of epigastric substernal chest pain relieved by aspirin and nitroglycerin. Upon examination, the patient's vital signs were as follows: blood pressure of 150/90 mmHg, heart rate of 85 beats per minute, respiratory rate of 18 breaths per minute, and oxygen saturation of 97% on room air. An echocardiogram revealed severe AS with an aortic valve area of 0.7 cm² and a mean valve gradient of 56 mmHg. Cardiac catheterization showed no significant coronary artery disease. Given her significant comorbidities and high surgical risk, TAVR was deemed the appropriate intervention. After informed consent was obtained, she was scheduled for the procedure.

During the TAVR procedure, immediately after valve deployment, the anesthesiologist noted that the right radial arterial line was not recording any waveform, and there was no palpable pulse in the right arm. Physical examination revealed the right arm was cyanotic, with pain, paresthesia, and an absence of the radial pulse. The right upper limb demonstrated profound hypoperfusion, marked by cyanosis from the elbow to the fingertips. The arm was cold to the touch, with absent radial and ulnar pulses. Doppler sonography indicated normal blood flow in the brachial artery but identified thrombotic material in both the ulnar and radial arteries beyond the brachial bifurcation. Crucially, no Doppler signal was detected from the proximal segments of these arteries to the periphery, underscoring the severity of the occlusion.

The patient was diagnosed with acute arterial occlusion of her right radial artery. The suspected source was a tissue fragment embolized to the arm during the TAVR procedure. An aspiration thrombectomy via groin access was performed, successfully restoring blood flow to the arm. It was hypothesized that she experienced an embolic event where a thrombus formed during TAVR valve deployment.

**Figure 1:**
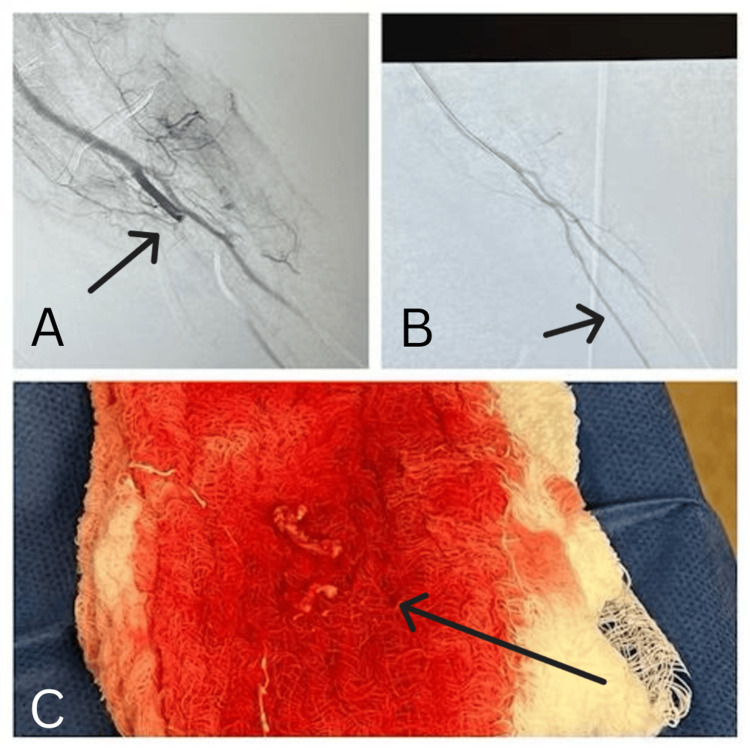
Digital subtraction angiography of the right upper limb (A) revealed significant thrombotic occlusions in the radial artery, with the brachial artery maintaining normal blood flow. Thrombus aspiration was performed using an Eliminate catheter (B), resulting in the prompt re-establishment of blood flow in the radial artery. The procedure successfully extracted the thrombus specimen, as evidenced in image C.

Digital subtraction angiography of the right upper limb revealed a significant thrombotic occlusion in the radial artery, although the brachial artery maintained normal blood flow. Thrombus aspiration was performed using the Eliminate catheter (Terumo, Tokyo, Japan), resulting in the immediate re-establishment of blood flow in the right radial artery. The procedure effectively extracted the thrombus specimen, restoring perfusion to the affected limb segments (Figure 1).

The patient's post-procedure recovery was smooth and complication-free. She was discharged with full functionality in her right hand. At the four-week follow-up, she remained symptom-free, and clinical examination of the right upper limb was normal. Doppler sonography showed excellent triphasic flow in both the brachial and radial arteries, along with retrograde filling of the ulnar artery.

## Discussion

In this case report, the patient experienced an acute thromboembolic occlusion in her right upper limb during the TAVR procedure. Following a thorough evaluation, the embolic event was attributed to the deployment of the TAVR valve.

Treatment options for aortic valve disease include TAVR and SAVR. For high-risk patients, TAVR has become the preferred treatment due to its minimally invasive nature [6]. Despite its benefits, TAVR has drawbacks such as PVL, conduction issues, and cerebrovascular events, occurring in 1% to 5% of cases [7]. Acute thromboembolic occlusion of the radial artery is rare, but stroke and TIA are more common cerebrovascular complications [9]. Most research on TAVR focuses on the link to stroke, as thrombi from the left heart often travel to the brain, though they can form in any channel during valve deployment.

Several factors contribute to embolic obstruction during TAVR. Catheters and valves can dislodge plaque or calcified material from the artery wall or valve, which may then enter the bloodstream and block a vessel. Vascular manipulation during the procedure can also activate blood clotting mechanisms, leading to clot formation and arterial blockage [6]. Additionally, temporary hemodynamic changes from valve implantation may increase embolism risk in the heart and major arteries [10]. Inflammatory responses induced by TAVR, both localized and systemic, can elevate thrombosis risk. Underlying conditions like hypertension, coagulation disorders, and prior cardiovascular disease further increase the likelihood of post-TAVR embolism [6,10].

The main treatment objective for acute arterial obstruction is restoring blood flow through the affected artery. While pharmacological methods like thrombolysis or anticoagulation can be used, they often yield poor results as complete flow restoration is challenging [6]. Systemic thrombolysis also carries a higher risk of bleeding. Traditionally, acute upper limb ischemia was treated surgically, but endovascular approaches such as mechanical thrombectomy and thrombolytic drug injections (e.g., reteplase and urokinase) have become more common. Mechanical thrombectomy is now the primary treatment for acute upper limb ischemia, though it carries risks like brachial artery dissection, bleeding, thrombosis, and pseudoaneurysm [6,10].

Cerebral embolic protection (CEP) devices aim to capture debris during TAVR and reduce stroke risk. However, recent research indicates that CEP does not significantly impact the incidence of periprocedural stroke in patients undergoing transfemoral TAVR. The 95% confidence interval of the study suggests it is not entirely possible to rule out the potential benefits of CEP in TAVR [10].

## Conclusions

This case underscores the rare but serious complication of acute thromboembolic occlusion of the radial artery during TAVR. While TAVR has become the preferred treatment for severe aortic stenosis due to its minimally invasive nature, it is not without risks. The potential for embolic events, such as stroke and limb ischemia, emphasizes the need for vigilance and prompt management of complications. The variability in the presentation of embolic events suggests that further research is necessary to fully understand and mitigate these risks. Investigating the mechanisms and prevention strategies for thromboembolic complications during TAVR is crucial for improving patient outcomes and safety. This case highlights the importance of vigilant intraoperative monitoring and rapid intervention to manage unforeseen complications effectively. Future studies should focus on exploring strategies to reduce embolic risks associated with TAVR, including the use of embolic protection devices. By advancing our understanding and management of these complications, we can enhance the safety and efficacy of TAVR procedures.
